# Temperature Effects on Toxicokinetics of Organic Chemicals in Aquatic Organisms

**DOI:** 10.1021/acs.est.5c00668

**Published:** 2025-10-28

**Authors:** Lea Grenc, Paul J. Van den Brink, A. Jan Hendriks

**Affiliations:** 1 Department of Environmental Science, 6029 Radboud University Nijmegen, Heyendaalseweg 135, Nijmegen, Gelderland 6525, The Netherlands; 2 Aquatic Ecology and Water Quality Management Group, Wageningen University, Droevendaalsesteeg 3a, Wageningen 6708PB, The Netherlands

**Keywords:** toxicokinetics, uptake, elimination, temperature, aquatic organisms, organic chemicals

## Abstract

Global temperature changes have implications for the toxicokinetics (TK) of organic chemicals in aquatic organisms. Using the Arrhenius equation, we analyzed a comprehensive data set of toxicokinetic parameters that included a wide range of aquatic animal species and organic chemicals to quantify how temperature influences uptake and elimination rate constants. Our findings show that higher temperatures lead to increased rate constants for both uptake and elimination across all of the tested organisms and chemicals. As the Arrhenius slopes for uptake and elimination are similar, the bioconcentration factor is largely independent of the temperature. Adjusting for the organism’s mass did not result in improved model fits. Furthermore, the octanol–water distribution coefficient (*D*_ow_) was incorporated for a chemical-specific analysis. Compounds with higher *D*_ow_ values exhibited higher uptake rate constants and lower elimination rate constants compared to those with lower *D*_ow_ values. Despite these chemical-specific nuances, the overarching trend of a temperature-dependent rate constant increase remained evident. These insights contribute to a better mechanistic understanding of the effects of temperature changes on the TK of organic chemicals in aquatic ecosystems.

## Graphical abstract

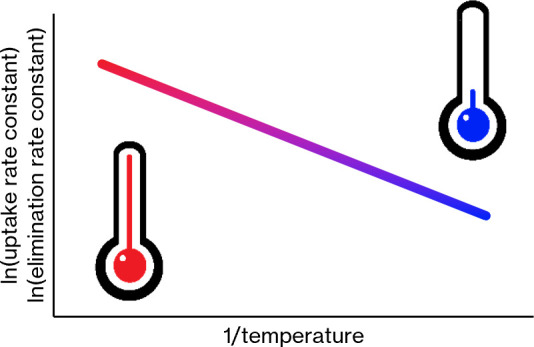

## 1 Introduction

Global warming has been causing changes in global temperatures.^1^ The ocean surface temperatures have already increased by 0.88 °C between 1850–1900 and 2011–2020 and have been projected to increase by another 0.86–2.89 °C between 1995–2014 and 2081–2100.^2^ Water temperatures have also been increasing in rivers (up to 1 °C per decade) and lakes (up to 0.45 °C per decade).^3^ Furthermore, heat waves are getting more frequent, more intense, and longer and will continue to do so,^4^ causing additional temperature increases in waterbodies. Aquatic organisms can have strong responses to environmental changes.^3^ Ectotherms, organisms whose body temperature regulation depends on external factors, are known to be affected by changes in the temperature. An increase in temperature affects the metabolic rates of cells and their size,^5,6^ affects population growth,^7^ can lead to higher mortality rates,^8^ and even results in community- and ecosystem-level impacts,^9^ particularly as a consequence of heat waves.

Anthropogenic activities cause pollution, including the release of organic pollutants. Most of them are micropollutants, which raise particular concerns because they can have significant biological effects due to their persistence, bioaccumulation potential, and toxic properties, despite often being present in the environment in low concentrations.^10,11^ The compounding effect of climate change and other stressors, including pollution, can already be observed.^12^ Increases in temperature most of the time lead to increased toxicity.^13–15^ However, some chemicals, such as pyrethroids, show decreased toxicity with rising temperatures.^16,17^ To effectively interpret and predict the temperature dependence of toxicity, it is crucial to understand how temperature impacts the kinetics and dynamics of substances in organisms. Toxicity is determined by two main factors: toxicokinetics (TK), which encompasses the uptake, distribution, metabolism, and elimination of toxicants, and toxicodynamics (TD), which involves the effects of these toxicants on biological systems. While the effects of temperature on various aspects of TK, such as uptake and elimination, have been explored, much of the existing research consists of individual studies focusing on a specific substance or species.^18–20^

This study aims to relate uptake and elimination rate constants of organic chemicals in aquatic animals to the temperature. To that end, we collected data from databases and the literature and fitted these to an Arrhenius equation. We also examined the relationship of these rate constants with the mass (*m*) of the animal and the octanol–water distribution coefficient (*D*_ow_) to reduce variability not caused by temperature. The outcomes will enable a risk assessment for organic chemicals and species, which includes the influence of climate change. This will make accumulation and toxicity models, such as the Optimal Modeling for Ecotoxicological Applications (OMEGA),^21^ the General Unified Threshold Model of Survival (GUTS),^22^ and many other models more robust and applicable in different environmental scenarios.

We hypothesized that both uptake and elimination rate constants increase with temperature, with the uptake rate constants rising more rapidly than the elimination rate constants, as organisms tend to be more sensitive to chemicals at higher temperatures. We also expected that correcting for organisms’ mass would improve the fit because physiological processes generally scale with body size. Furthermore, we anticipated that temperature impacts on toxicokinetics would differ among chemicals, partly influenced by *D*_ow_. Lastly, we expected that species-specific responses would not always be predictable solely on the basis of these factors.

## 2 Materials and Methods

### 2.1 Theory

The Arrhenius equation is widely used to describe the temperature dependence in (bio)­chemical processes. In the context of toxicokinetics, uptake and elimination are predominantly driven by transport processes such as diffusion and advection.^19^ Therefore, we used the Arrhenius equation to establish an exponential relation between temperature and toxicokinetic rate constants:^23^

$$k=A\times e^{-E_{A}/RT}$$ 1

In this equation, *k* stands for the uptake or elimination rate constant (L/kg day^–1^ or day^–1^, respectively) at temperature *T*. *A* denotes the frequency factor (or the pre-exponential factor or Arrhenius factor, in L/kg day^–1^ or day^–1^ for uptake and elimination rate constants, respectively). *E*_A_ represents the activation energy in J mol^–1^, *R* is the ideal gas constant (8.31 J K^–1^ mol^–1^), and *T* signifies the temperature in Kelvin.

Rate constants can be adjusted to account for the mass of organisms using a relation that has a mechanistic interpretation and has been previously employed to relate size to physiological^6^ and toxicokinetic processes.^24,25^ Rates *F* (kg/animal/day) generally increase with mass *m* (kg) to the power of 3/4 so that rate constants *k* (kg/kg/day) decrease with mass to the power of − 14 according to *k* ∼ *F*/*m* ∼ *m* ^3/4^/*m* = *m* ^–1/4^. So, differences between organisms can, in principle, be normalized by dividing uncorrected rate constants *k* by *m* ^–1/4^ to arrive at mass-corrected rate constants k*, as follows:

$$k^{*}=\frac{k}{m^{-1/4}}$$ 2

In this equation, *k* stands for the uncorrected uptake or elimination rate constant (L/kg day^–1^ or day^–1^, respectively) and *m* denotes the mass of an organism (kg). The corrected rate constant k* represents the uptake or elimination rate constant normalized to a 1 kg organism (L/kg kg^1/4^ day^–1^ or kg^1/4^ day^–1^, respectively). So, k* effectively expresses the coefficient of the allometric relationship *k* = k* × *m* ^–1/4^.^24^

To cover variability between substances, we also analyzed the relationship between the TK parameters and the *D*_ow_. Specifically, we considered *D*_ow_ values for chemicals containing ionizable atoms, determining these values using MarvinSketch (https://chemaxon.com/marvin). Plotting uptake and elimination rate constants as a function of *D*_ow_ allowed us to explore the correlation between *D*_ow_ values and the respective rate constants.

### 2.2 Data Collection and Treatment

Data on uptake and elimination rate constants were gathered from two primary sources, the MOSAIC_bioacc_ platform (https://lbbe-shiny.univ-lyon1.fr/mosaic-bioacc/) and Hendriks et al.^24^ The MOSAIC_bioacc_ platform is a dedicated web tool designed to calculate bioaccumulation metrics using toxicokinetic models. We used toxicokinetic data from the MOSAIC_bioacc_ platform and obtained the connected temperature data from the source articles. Hendriks et al.,^24^ on the other hand, developed a comprehensive model for the accumulation kinetics of organic substances, considering factors such as the octanol–water partition ratio (*K*_ow_) ecological and biological parameters, including weight, lipid content, and trophic level of the species but not temperature.^24^ Additionally, a manual search on Google Scholar was conducted using keywords “toxicokinetics”, “aquatic”, “uptake”, and/or “elimination” to collect additional data. To compile comprehensive information, all data source articles were thoroughly reviewed to identify gaps primarily related to temperature data. Only data sets where temperature conditions remained consistent throughout the experiment were included. In cases where multiple data points were available for the same species and chemical at identical temperatures (with temperature values rounded to the nearest whole number), the duplicate entries were averaged. This approach was employed to prevent potential bias or the overweighting of specific combinations in the analysis.

For the analysis, we focused on organic substances in aquatic organisms, including crustaceans, mollusks, insects, and fish. In total, 530 and 958 data points were included for the uptake and elimination rate constants, respectively.

We applied a linear version of the Arrhenius equation to fit the data:

$$\ln\;k=\ln\;A-\frac{E_{A}}{R}\times \frac{1}{T}$$ 3

with the uptake (L kg^–1^ day^–1^) or elimination (day^–1^) rate constant *k* and the inverse temperature 1/*T* (1/K). The natural logs of the rate constants were plotted as a function of the inverse of the temperature to determine the slope *E*_A_/*R* (K) and intercept ln *A* (/) of the linear regression, implemented in Python 3.10. The *p*-value of the regression was also calculated, with *p* < 0.05 considered statistically significant.

For the mass-corrected rate constants, averages from body mass data of the same species, taken from other studies, were employed in instances in which the body mass for a particular species was missing in the primary study. In cases where the life stage was specified, the average body mass of that specific life stage was preferably used.

To obtain reliable regressions, only data sets with a minimum of four different temperatures for either uptake or elimination rate constants across all sources were considered when focusing exclusively on chemical- and species-specific analysis. This was implemented to mitigate the potential impact of insufficient data on the accuracy and reliability of trendlines generated from the data set. While this reduced taxonomic diversity, the resulting data set still includes species with a broad range of physiological traits.

## 3 Results and Discussion

### 3.1 Mass-Uncorrected Rate Constants

Most data for uptake and elimination rate constants were available for taxa belonging to Insecta, Osteichthyes, and Crustacea. Studies usually evaluated chemicals, including PCBs, phosphor biocides, and halo biocides, for uptake rate constants, while elimination rate constants were mostly assessed for PCBs, PAHs, and halobiocides (Table 1).

**Table 1:** Number of Species (with the Number of Data Points) for which Uptake and Elimination Rate Constant Data Were Available, Categorized by Taxon (a), and the Number of Chemicals (with the Number of Data Points) for which Uptake and Elimination Rate Constant Data Were Available, Categorized by Chemical Group (b)

(a)			(b)		
taxon	uptake	elimination	chemicals	uptake	elimination
Amphibia	0 (0)	1 (2)	bromo diphenyl ethers (BDE)	3 (3)	13 (27)
Bivalvia	3 (50)	10 (122)			
Camarodonta	0 (0)	1 (1)	chloronaphthalenes	9 (9)	9 (10)
Copepoda	0 (0)	1 (1)	halobiocides	11 (32)	19 (72)
Crustacea	13 (211)	24 (276)	halobenzenes	12 (34)	13 (40)
Gastropoda	4 (13)	12 (21)	polycyclic aromatic hydrocarbons (PAHs)	11 (56)	19 (150)
Insecta	18 (49)	18 (50)			
Oligochaeta	2 (6)	3 (40)	polychlorinated biphenyls (PCBs)	49 (129)	91 (241)
Osteichthyes	17 (195)	32 (433)			
Polychaeta	1 (4)	3 (10)	phosphorbiocides	13 (38)	17 (49)
Pycnogonida	0 (0)	1 (1)	others	74 (229)	141 (369)
Thecostraca	0 (0)	1 (1)	Total	182 (530)	322 (958)
Total	58 (530)	107 (958)			

The mass-uncorrected uptake rate constants were statistically significantly correlated to temperature (*p* < 0.05). As shown in the left panel of Figure 1, the natural logarithm of the uptake rate constant increases with an increase in temperature (note that 1/*T* is displayed on the *x*-axis). This correlation is linear and statistically significant.

**Figure 1:**
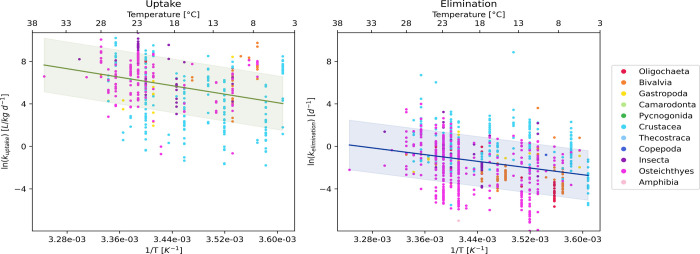
Uncorrected uptake and elimination rate constants for all species fitted to the linear version of the Arrhenius equation.

The analysis of the uncorrected elimination rate constants also demonstrated a significant correlation with temperature (*p* < 0.05). As depicted in the right panel of Figure 1, the natural logarithm of the elimination rate constant also increases with a rising temperature.

Our multisubstance and multispecies analyses align with previous single-substance and single-species studies showing that the uptake and elimination rate constants increase exponentially with temperature,^19,20^ as reflected by a trend in log-transformed data. To provide further context, several studies explicitly investigating temperature’s impact on TK have reported similar trends. Jimenez et al.^26^ reported higher uptake and elimination rate constants of benzo­(*a*)­pyrene at higher temperatures. Similarly, Huang et al.^18^ observed increased uptake and elimination rate constants for imidacloprid and flupyradifurone with rising temperature, although the rate constants did not increase to the same extent. Maruya et al.^27^ found slower elimination of toxaphene at lower temperatures, indicating temperature-dependent variations. Edgren et al.^28^ observed a doubling in PCB accumulation with a 10 K increase, but elimination rate constants were unaffected by temperature. Brown et al.^29^ observed faster elimination of PCBs and PBDEs at higher temperatures, which is consistent with our findings. These studies support the conclusion that the temperature increases toxicokinetic rate constants across different species and chemicals. Moreover, the similarity in Arrhenius slopes observed for uptake and elimination suggests that these processes change with temperature at approximately the same rate, making bioconcentration factors largely temperature-independent. Similar conclusions were reported by Brown et al.^29^ and Raths et al.^20^

### 3.2 Mass-Corrected Rate Constants

The mass-corrected uptake and elimination rate constant analysis also showed a statistically significant correlation of both rate constants with temperature (*p* < 0.05) (Figure 2).

**Figure 2:**
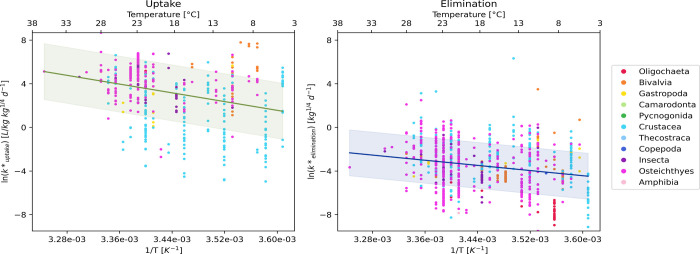
Mass-corrected uptake and elimination rate constants for all species fitted to the linear version of the Arrhenius equation.

The coefficients of variation (CV) (Table 2) show that the mass-corrected rate constants for uptake had slightly lower CVs for both the slope and intercept compared with the uncorrected rate constants. In contrast, for elimination, the corrected rate constants exhibited marginally higher CVs for both parameters, with the differences being minimal. These findings suggest that correcting for body size slightly reduces variability in uptake parameters while having a less pronounced effect on elimination parameters. Although small, this improvement aligns with previous findings that emphasized the importance of size in TK.^30,31^ Additionally, Rubach et al.^32^ tested both adults and juveniles of two species and highlighted that physiological factors, such as the increase in body size, negatively influence uptake and elimination rate constants.

**Table 2:** Parameters of Equation 3 for Uptake and Elimination Rate Constants ± SD (CV [%]) [95% CI], Comparing Models Uncorrected and Corrected for Mass

model	rate constants	slope [K]	intercept [/]	*R* ^2^	*p*-value
uncorrected	uptake	–10,000 ± 1496 (15.0) [−7060 −12,939]	40.11 ± 5.14 (12.8)	0.093	<10^–4^
	elimination	–7888 ± 1081 (13.7) [−5766 −10,009]	25.73 ± 3.73 (14.5)	0.061	<10^–4^
corrected for mass	uptake	–10,026 ± 1516 (15.1) [−7046 −13,005]	37.65 ± 5.22 (13.9)	0.091	<10^–4^
	elimination	–5905 ± 974 (16.5) [−3994 −7816]	16.83 ± 3.36 (20.0)	0.043	<10^–4^

When examining the *R* ^2^ and *p*-values (Table 2), differences between mass-corrected and uncorrected fits were observed. For uptake rate constants, the *R* ^2^ value slightly decreased from 0.093 for uncorrected data to 0.091 for mass-corrected data, indicating a marginally poorer fit after mass correction. Similarly, the *p*-values remained highly significant (*p* < 0.0001 for uncorrected and corrected), further confirming the strong correlation between temperature and uptake rate constants in both cases. In the case of elimination rate constants, the *R* ^2^ value for uncorrected data was 0.06, while the mass-corrected data showed a marginal decrease in *R* ^2^ to 0.04. While these *R* ^2^ values may appear low, this is expected given the high taxonomic and physiological diversity of the data set. Differences in biotransformation capabilities, water exchange surfaces, and uptake and elimination mechanisms likely contribute to the observed variability. Despite this heterogeneity, the significance of the relationships suggests that temperature consistently influences TK across taxa. Both elimination rate constant models exhibit highly significant *p*-values (*p* < 0.0001 for uncorrected and corrected), underscoring the strong temperature dependence of these rate constants. While the changes in *R* ^2^ are not substantial in the present data set, Hendriks et al.^24^ reported that the organism mass explained approximately 39 and 70% of the variability in uptake and elimination rate constants, respectively. Rubach et al.^32^ found similar values for the insecticide chlorpyrifos, with length and dry mass explaining 47 and 38% of the variation in uptake rate constants and 55 and 34% of the variation in elimination rate constants of 15 invertebrate species, respectively.

These findings shed light on the role of the organism size on TK. The mass correction approach did not yield substantially better fits for uptake or elimination rate constants. The 95% confidence intervals for the slopes of uncorrected and mass-corrected models overlap substantially (Table 2), indicating no statistically significant difference between the slopes. However, the mass-corrected uptake slope was −10,026 ± 1516 K, which is slightly steeper than the average slope of −7195 K reported by Gillooly et al.^6^ for physiological processes. While it falls just outside their reported range (−8588 to −4758 K), this proximity still supports the idea that uptake processes could scale with both body size and temperature in a manner similar to that of other physiological rates. For elimination, the mass-corrected slope in our study was less steep at −5905 ± 974 K, indicating that elimination rate constants in the studied species are less temperature-sensitive, consistent with findings by Huang et al.^19^ This could be due to physiological processes, such as biotransformation and active excretion. Nevertheless, these overall trends are consistent with the universal temperature dependence observed in physiological processes. The parallels between mass-corrected toxicokinetic rate constants and mass-specific rate constants of physiological processes suggest that temperature-driven changes in uptake and elimination are caused by temperature-driven changes in physiological processes, determining the transport and transformation of chemicals, such as ventilation, ingestion, excretion, egestion, and enzyme kinetics.

### 3.3 Chemical-Specific Responses

The uptake rate constants generally increase with increasing temperature for each *D*_ow_ value representing a single substance (Figure 3). The slopes of the regression lines are not notably different based on the *D*_ow_ (*p* > 0.05); however, those chemicals with lower *D*_ow_ values generally have lower intercepts compared to those with higher *D*_ow_ values.

**Figure 3:**
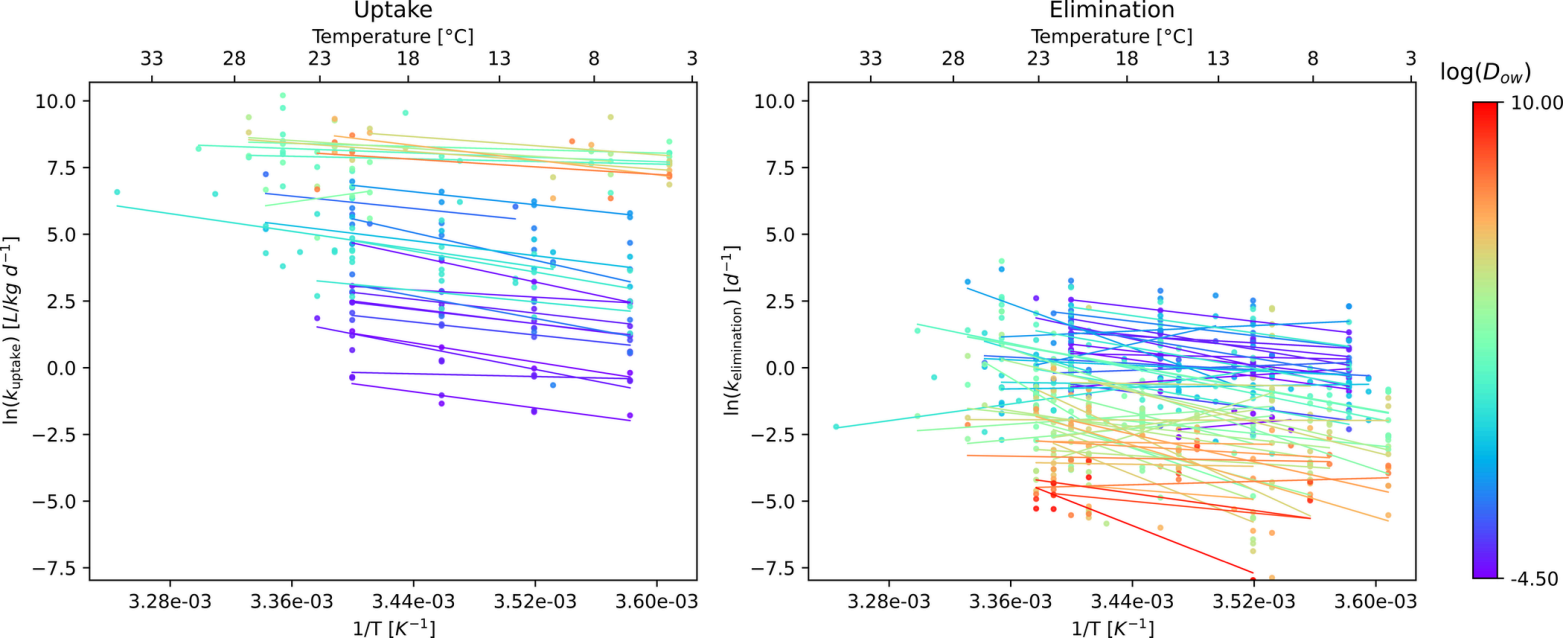
Uptake and elimination rate constants for all species and different chemicals are based on their *D*_ow_ values, ranging from low *D*_ow_ values (blue) to high *D*_ow_ values (red).

The right panel of Figure 3 presents the elimination rate constants and their relationship with the temperature and *D*_ow_. Like the uptake rate constants, the elimination rate constants also increase significantly with increasing temperature, with the *D*_ow_ values affecting the correlation. However, in the case of elimination rate constants, the chemicals with lower *D*_ow_ values generally have higher elimination rate constants compared to those with higher *D*_ow_ values. An increase in uptake and a decrease in elimination with increasing hydrophobicity have been demonstrated for inert chemicals before, albeit for octanol–water partition ratios (*K*_ow_) rather than distribution ratios (*D*_ow_).^24^

Both the uptake and elimination graphs demonstrate that the correlation between the temperature and the rate constants is higher for chemicals with the same *D*_ow_ values compared to the ones from Figure 1. Exceptions to these trends could be attributed to the limited data available and the differences in species tested. Notably, most data in this section come from studies on crustaceans. The dominance of data from a single taxon likely influences the observed trends, limiting the applicability of the results to other taxonomic groups. This highlights the need for additional data on a broader range of taxa to better understand the temperature effects on aquatic organisms.

The similarity of Arrhenius slopes for toxicokinetic rate constants across different chemicals and taxa, and their alignment with those observed for general physiological processes (see Section 3.2), suggests the potential for a universal temperature correction factor. If confirmed more broadly, then this would imply that additional chemical-specific temperature testing might become unnecessary as extrapolation based on a single measurement and a general physiological slope could suffice. This finding highlights the strength of our generalized approach and presents a promising direction for streamlining future risk assessments.

### 3.4 Species-Specific Responses

The uptake rate constants of various chemicals by *Gammarus pulex* (*G. pulex*), the species with the most comprehensive data set, show a statistically significant (*p* < 0.05) correlation with temperature (Figure 4). All data shown in Figure 4 originate from a single study from Raths et al.,^20^ which tested multiple chemicals under controlled conditions across a temperature gradient. This allows one to examine temperature effects while variability in experimental design, species traits, and exposure conditions is minimal. In this data set, uptake rate constants increase with rising temperature. This trend is consistent across chemicals with varying *D*_ow_ values. Similar to the uptake rate constants, the elimination rate constants also display a positive correlation with increasing temperature. Similar to multispecies results, differences in intercepts are, however, not consistently explained by the *D*_ow_ for the *G. pulex* data. These deviations could be explained by the chemical’s size influencing uptake and by biotransformation affecting elimination.

**Figure 4:**
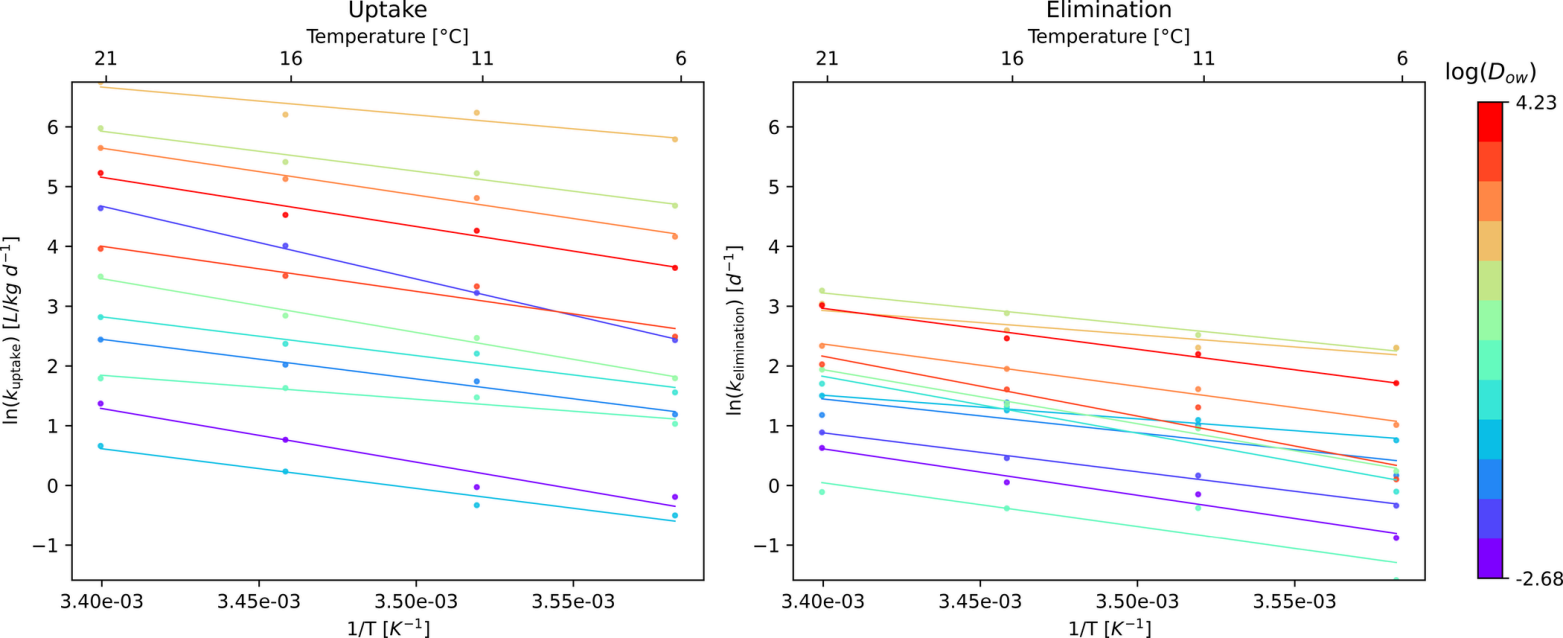
Uptake and elimination rate constants for *Gammarus pulex* with chemicals separated based on their *D*_ow_ values.

Our observations are supported by Huang et al.^18^ and Mangold-Döring et al.,^19^ who provided further context on the temperature-dependent TK in *G. pulex*. Huang et al.^18^ reported that the impact of temperature on uptake and elimination rate constants varies between different insecticides, illustrating that temperature effects are crucial for understanding chemical behavior. While Huang et al.^18^ do not explain these differences, our findings also show variability in slopes between chemicals, suggesting that temperature effects on TK may be partly chemical-specific. Mangold-Döring et al.^19^ demonstrated that incorporating temperature into TK models significantly improves the accuracy of predicting internal chemical concentrations, which corresponds with our findings.

In summary, our results show that temperature consistently increases uptake and elimination rate constants across a wide range of species and chemicals, while bioconcentration factors remain largely temperature-independent due to similar changes in uptake and elimination. Adjusting for the organism mass only slightly improves model fits. Although chemical-specific properties, such as *D*_ow_, influence the absolute rates, the general pattern of temperature-driven increases remains consistent. These findings provide a mechanistic basis for incorporating temperature effects into TK models across multiple species and compounds.

## Supplementary Material



## Notes

Spreadsheet with uptake and elimination data (XLSX)
